# Editorial Comment: Long-term outcomes and risks factors for failure of intradetrusor onabotulinumtoxin A injections for the treatment of refractory neurogenic detrusor overactivity

**DOI:** 10.1590/S1677-5538.IBJU.2021.02.03

**Published:** 2021-02-03

**Authors:** Marcio Augusto Averbeck

**Affiliations:** 1 Hospital Moinhos de Vento Unidade de Videourodinâmica Porto Alegre RS Brasil Chefe de Neuro-Urologia, Unidade de Videourodinâmica, Hospital Moinhos de Vento. Porto Alegre, RS, Brasil.

Charles Joussain ^1^, Mélanie Popoff ^2^, Véronique Phé ^3^, Alexia Even ^2^, Pierre-Olivier Bosset ^4^, Sandra Pottier ^5^, et al.

## COMMENT

First-line treatment of neurogenic detrusor overactivity (NDO) has been traditionally based on oral antimuscarinics. Conservative treatment failure, either due to antimuscarinic adverse events or to a lack of efficacy, usually leads to intravesical injection of botulinum toxin type A or more complex procedures such as bladder augmentation. Intradetrusor injection of OnabotulinumtoxinA (Botox®) is licensed worldwide and recommended as a second line therapy for the treatment of urinary incontinence due to NDO after failure of antimuscarinics. However, only very few studies assessed the long––term efficacy of Botox® in patients with refractory NDO. Joussain et al. performed a retrospective analysis of consecutive neurological patients using intermittent catheterization (IC) who had received Botox® injections between January 2001 and September 2013. A total of 292 patients (170 males, 122 females) were included, of whom 85% fell into the category of traumatic spinal cord injury, 11% had multiple sclerosis (MS) and 5% had other conditions. Average age at baseline was 40 years. Mean follow-up was 4.2 ± 1.8 years. At last follow-up, mean number of Botox® injections was 9.7 ± 4.7 per patient. The mean delay between two Botox® injections was 6.5 ± 1 months with 300U and 5.1 ± 1.4 months with 200U. After an initial 200U dose, a switch into a 300U dose had to be used in 93 patients (49.2%) due to premature recurrence of UI. Overall, 81% were still being treated after 3 years, 71% after 5 years and 61% after 7 years. Failure rate was 13% after 3 years, 22% after 5 years and 29% after 7 years of follow-up. Risks of failure were cited as severe detrusor overactivity at baseline, high maximum detrusor pressure, presence of urinary incontinence before the first injection, number of febrile urinary tract infections, low bladder compliance and renal ultra-sound abnormalities. An interesting finding was the withdrawal rate after 7 years of follow-up (11.3%), primarily due to IC-related difficulties, which prevailed among MS patients, accounting for 54.4% of all IC-related withdrawals. This observation reinforces the need for a better education on IC among neuro-urological patients, in particular for MS patients.

quality of life assessments, detailed description of inclusion and exclusion criteria, surgical technique and post-operative complications. Perhaps an international registry/working group may overcome these inherent limitations and increase the level of evidence in the near future.

